# Experimental Study on Repairing the Mechanical Characteristics of Oil-Contaminated Silty Clay in Ancient Dike with Modified Lime Mortar

**DOI:** 10.3390/ma16093449

**Published:** 2023-04-28

**Authors:** Jianfen Zhou, Zhiyong Dong, Yinzhen Dong, Xiaohui He, Hongmei Wu, Bin Chen, Qian Mao

**Affiliations:** 1 College of Civil Engineering, Zhejiang University of Technology, Hangzhou 310014, China; zhoujf@zjweu.edu.cn; 2 Key Laboratory for Technology in Rural Water Management of Zhejiang Province, Zhejiang University of Water Resources and Electric Power, Hangzhou 310018, China; 3 Lanxi Town Planning Office, Jinhua 321100, China; 4 Lanxi Water Affairs Bureau, Jinhua 321100, China

**Keywords:** flood-controlled ancient dike, silty clay, oil pollution, mechanical characteristic of MLM soil, MLM pressure grouting, seepage coefficient of MLM soil

## Abstract

Flood-controlled ancient dikes play a significant role in flood control and have received widespread attention as historical and cultural symbols. Flood-controlled ancient dikes often undergo disasters, and research on their repair is receiving increasing attention from experts and scholars. This article studies the control of seepage and bank slope instability in flood-controlled ancient dikes. Starting from the repair of ancient dike materials, three types of work are carried out: a test of soil’s mechanical properties, finite element numerical simulation, and repair technology research. The research results show that the soil of the ancient dike site has hardened after being contaminated with waste oil from catering. The strength index of the ancient dike soil decreases and shows brittleness when the water content is 15% and the oil content exceeds 6%. The strength index and permeability coefficient of oil-contaminated soil improved using modified lime mortar (MLM), which was achieved using the method of MLM to repair oil contaminated soil. When the MLM content was 10% and the oil content was 6%, the friction angle of the soil sample reached its maximum value. When the MLM content was the same, the higher the density of the soil sample, the greater the friction angle and cohesion and the smaller the permeability coefficient. Establishing a finite element numerical model, through comparative analysis, it was found that after MLM remediation of oil-contaminated soil, the extreme hydraulic gradient of the ancient dike decreased by 31.3%, and the extreme safety factor of the bank slope stability increased by 31.2%. MLM pressure grouting technology was used to improve the soil during the remediation of contaminated soil at the ancient dike site. Through on-site drilling inspection, the effective diffusion radius of MLM grouting was obtained, and the plane layout and grouting depth of MLM pressure grouting were determined. The on-site water injection permeability test showed that using MLM pressure grouting technology can effectively repair oil-contaminated soil in the ancient dike while reducing the permeability coefficient by 8–15%.

## 1. Introduction

The urbanization process in China has had a significant impact on the urban water system structure, and urban construction planning optimization based on flood control safety and historical water engineering protection is gradually becoming a research hotspot [1,2,3,4,5]. Many ancient embankments in southern China are adjacent to residential buildings, and rainwater and sewage pipelines are staggered. While ancient dike works play the role of flood control, they are affected by the leakage of waste oil from residents’ catering, resulting in changes in the nature of the soil mass of the flood-control dike, resulting in the aggravation of the damage of piping soil flow caused by flood scouring and changes in the soil strength, resulting in the change of the overall stability of the bank slope and even landslide damage. Lime is the main building material for ancient projects around the world. The repair of ancient dikes requires the use of lime, which has better compatibility with the materials of the original structure to be repaired. There are few reports on the remediation of oil-contaminated soil with lime. Previous research has mainly focused on two aspects: on the one hand, experimental research on the physical and mechanical properties of mineral oil-contaminated soil, and on the other hand, the research on lime-modified raw soil and the reparation of ancient building materials.

The mineral oil of common contaminated soil includes oil, gasoline, diesel, and gasoline, and the contaminated soil includes cohesive soil, fine sand, silty clay, etc. Oil-contaminated soil samples were prepared by mixing the residual soil and oil and were tested in the laboratory. The results showed that oil pollution led to the deterioration of the geotechnical properties of the soil samples. Compared with raw soil, its maximum dry density, optimum water content, permeability, and shear strength decreased [6,7,8]. The particle size distribution and density of soil samples have a great impact on the mechanical properties and permeability of oil-contaminated clayey silt. With the increase in oil content, the friction angle of soil samples decreases, the cohesion does not change significantly, and the permeability coefficient increases significantly [9]. After the gravel is polluted by oil, with the increase in oil content, the friction angle decreases and the cohesion increases significantly [10,11,12]. After the clay is polluted by gasoline, the pH value of the clay decreases significantly and the conductivity increases. Additionally, at first, with the increase in gasoline content, the friction angle slightly increased and the cohesion decreased significantly. However, with the increase in the lead content in gasoline, the friction angle and cohesion decreased [13,14]. Another study investigated the effect of petroleum-derived contaminants on the permeability, cohesion, friction angle, and shear strength of fine sand. An increase in cohesion was observed for sand with up to 1% oil contamination, after which the cohesion began to decrease, which also resulted in the reduction in the permeability [15]. After diesel contaminated the loess, the permeability coefficient of the loess decreased with the increase in diesel content, and the compression modulus and unconfined compressive strength were almost unchanged [16,17,18]. Gasoline pollution poorly graded sand, low-plasticity clay, and silt, and decrease in the friction angle and an increase in the cohesion of the soils were found with an increase in contamination. Unconfined compressive strength of silty soil decreased with increasing gas oil percentage, which is different from low plasticity clay [19,20]. The oil content also affects the bearing-capacity characteristics of sandy loam soil and the settlement of the footing, and was also found to significantly reduce the load settlement and ultimate bearing capacity of the footing [21]. Through the direct shear test and bearing capacity test of strip shallow foundation, it is shown that the oil content reduces the shear strength and bearing capacity of sand to varying degrees [22]. Based on the above research, the physical and mechanical behavior of different types of soil polluted by different kinds of mineral oil is complex, which is quite different from the original soil’s properties. It is necessary to evaluate the changes in physical and mechanical properties of silty clay contaminated by oil and to analyze the impact of waste oil pollution on the stress–strain relationship, seepage characteristics, and microstructure characteristics of silty clay, providing basic data for the improvement in the contaminated soil.

Due to the special historical and cultural value of ancient dikes to be preserved during repair, the repair materials should be compatible with the raw materials, and lime should be the main material [23,24,25,26,27]. There are many experimental studies on adding lime and additives to change soil properties. Previous studies have obtained five properties of natural hydraulic lime mortar, namely the type of natural hydraulic lime [28,29,30]; the ratio of binder to aggregate [31]; the effect of the water–cement ratio on the fluidity of lime mortar [32,33]; the characteristics of aggregate, including composition, particle size, and distribution [34]; and the curing conditions [35]. The results show that when the ratio of binder to aggregate of lime mortar is 1:3, its mechanical properties can be improved and better carbonation can be obtained [36,37,38]. Lime and binder can be used to change the mechanical properties and durability of raw soil, and the modification effect of raw soil will increase with the increase in lime content [39,40,41,42,43,44].

Many scholars have studied the effects of physical and mechanical properties of soil on the seepage and bank-slope stability of flood control dikes. Based on the change in the microstructural arrangement of soil particles, the microscopic change in the permeability coefficient of soil is studied experimentally [45,46,47]. The coupling equation of seepage and erosion shows that the pore water pressure in soil increases until the hydraulic gradient is greater than the critical hydraulic gradient, and the fine particle phase migrates and loosens from the soil, resulting in deformation and instability [48,49,50,51]. Research on bank-slope stability has shown that numerical analysis methods provide an appropriate and effective tool to establish numerical models. Based on changes in the intrinsic properties of soil and experimental data, the bank-slope stability remains to be analyzed [52,53,54,55,56,57]. Current research rarely involved the characteristics of lime to improve the oil-contaminated soil, and carrying out relevant research is urgently needed to repair the soil to reinforce the ancient dike. 

## 2. Methodology and Materials

### 2.1. Methodology

**Figure 1:**
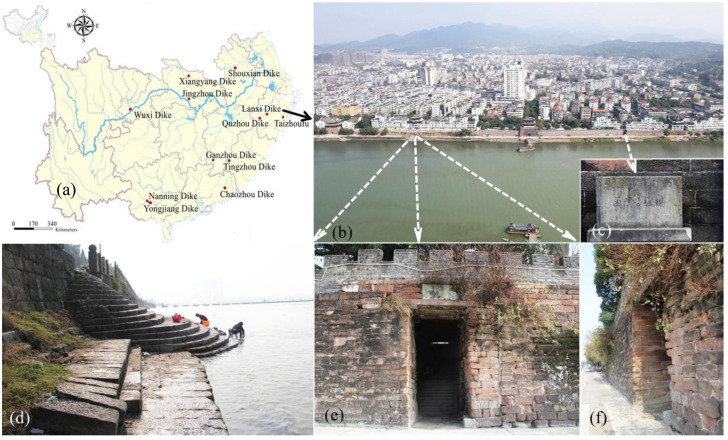
Characteristics of flood-controlled ancient dikes in South China. (**a**) Typical ancient dike distribution; (**b**) aerial view of Lanxi Ancient Dike; (**c**) ancient dike boundary stele; (**d**) ancient wharf; (**e**) city gate hole in ancient dike; (**f**) side view of ancient dike.

There are many flood-controlled ancient dikes in China. These ancient dikes still play a role in flood control, and at the same time, they are often protected cultural relics and landmark buildings in towns. Representative flood-controlled ancient dikes in southern China are all distributed in densely populated towns along rivers (in Figure 1a). Take the Lanxi ancient dike as an example, which is located at the intersection of Qujiang, Jinhua, and Lanjiang rivers, as shown in Figure 1b. The ancient dike was first built in 1512 AD and consists of a wharf and an ancient dike [58], as shown in Figure 1c,d. The ancient dike is built of red sandstone, and the city gate hole is the entrance and exit from the ancient wharf to the town, as shown in Figure 1e,f.

**Figure 2:**
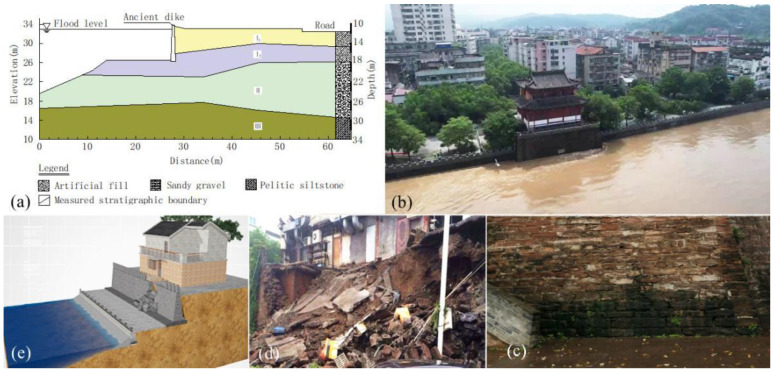
Damage to ancient dike. (**a**) Engineering geology of Lanxi ancient dike; (**b**) flood of Lanjiang river; (**c**) leakage; (**d**) oil pollution; (**e**) bank slope collapse.

Ancient dikes have suffered various forms of damage. The bank slope of Lanxi Ancient Dike is mainly filled with artificial soil, Figure 1a. During the rainy season, the confluence of river water often leads to a surge of river water (Figure 2b). In addition, rainfall and groundwater collection lead to leakage of the ancient dike wall, and waste oil from residential and catering pollutes the soil, as shown in Figure 2c,d. After the soil of the ancient dike was polluted by the waste oil from catering, the stability of the bank slope was reduced, and landslides and other damages occurred, as shown in Figure 2e.

**Figure 3:**
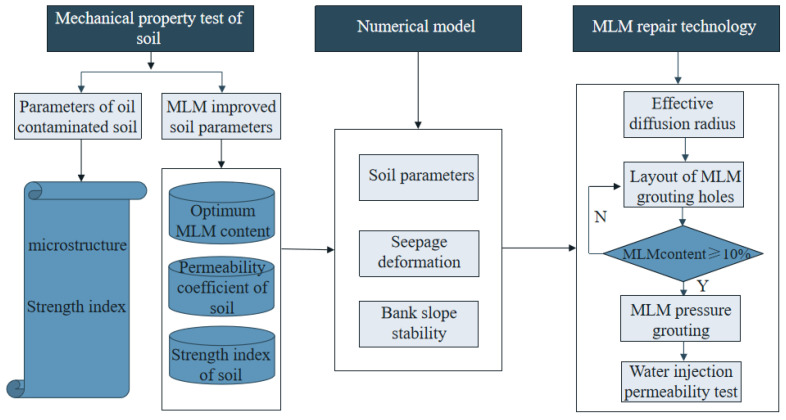
Flowchart of the proposed methodology.

Through experiments, the physical and mechanical parameters of soil were obtained, and changes in soil, oil content, and modified lime mortar (MLM) content on mechanical indicators were compared and analyzed. Then, the stability of flood-controlled ancient dike was studied using the finite element numerical analysis approach to take into account the intrinsic variability of the soil parameters. The research provides theoretical guidance for the study of ancient dike seepage, slope stability, and reinforcement measures, as shown in Figure 3. 

### 2.2. Materials

#### 2.2.1. Soil

**Figure 4:**
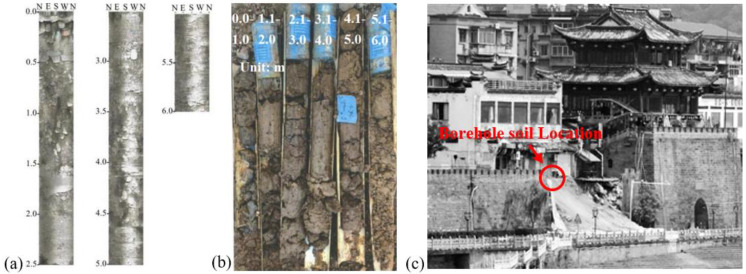
Soil sampling of a typical section of an ancient dike. (**a**) TV histogram of boreholes in strata I_1_ and I_2_; (**b**) bored soil core of soil layers I_1_ and I_2_; (**c**) bank slope collapse.

The soil for the test was taken from a typical section of the Lanxi Ancient Dike. Figure 4a,b is a typical section of the Lanxi dike. It follows that the ancient dike’s geology can be divided into three soil layers, namely newly filled layers I_1_ and I_2_ and sand gravel-filled layer II. The thickness of soil layers I_1_ and I_2_ is 2.5~4.5 m, the thickness of sand gravel layer II is 6.1~11.5 m, and the layer below layer II is the argillaceous silty sand layer. The soil of layer I_1_ is newly artificially filled soil, mainly composed of silty cohesive soil with mixed rubble and a small number of broken bricks in the middle. In some embankment sections, there are still brick wall structures of previous houses left. This layer of soil has poor uniformity, complex material composition, unstable properties, and poor impermeability. The filling soil of layer I_2_ is mainly composed of silty clay with sand gravel. Compared to layer I_1_, this layer has a relatively long filling history. The structure of this layer is moderately dense, with general uniformity. This layer of soil has medium water permeability. Layer II is composed of sandy gravel pebbles, mixed with silty clay, and is moderately dense with medium water permeability. The impact range of waste oil leakage from catering is mostly in layer I_1_ and layer I_2_, and the impact range of seepage deformation is in layer I_1_, layer I_2_, and layer II. The soil used in this test is taken from this range, and after drying and screening, the soil with a particle size of less than 2 mm is taken for the remolded soil test.

The ancient dike is adjacent to residential buildings and municipal facilities, and the bank slope often collapses suddenly, as shown in Figure 4c.

The dry density and moisture content of soil samples of soil layers I_1_, I_2_, and II were measured with the drying method, the specific gravity with the pycnometer method, and the liquid plastic limit and plastic limit of Ⅰ_1_ and Ⅰ_2_ soil samples with the combined liquid plastic limit method according to the Standard for Soil Test Methods (GB/T50123-2019), as shown in Table 1.

**Figure 5:**
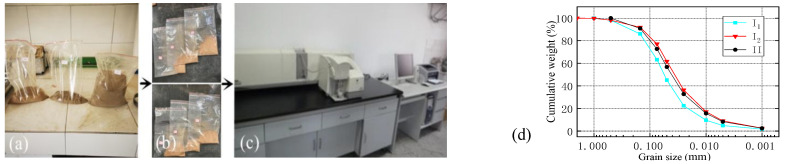
Particle size distribution test of soil sample. (**a**) Sandbagging at 0.075~2 mm; (**b**) fine particles less than 0.075 mm were packed separately; (**c**) laser particle size analyzer; (**d**) gradation curve of soil samples.

After the soil sample was fully air-dried, it was fully rolled with a rubber rod and sieved through a 2 mm sieve, and the sieve residue was stored in a sealed bag for standing by. In order to analyze the composition and distribution of soil particles, a 0.075 mm sieve was used to divide the soil samples into two groups: coarse particles with a diameter of 0.075~2 mm and fine particles with a diameter of less than 0.075 mm. The soil samples were put into a self-sealing bag for standby, as shown in Figure 5a. Then, 0.075~2 mm soil sample were sieved with fine screen, and fine particles smaller than 0.075 mm were analyzed with a laser particle size analyzer (MS2000) (Hangzhou, Zhejiang province, China), as shown in Figure 5b,c. The instrument can analyze particles of 0.02~2000 μm, adopt a scanning speed of 1000 times/s, and perform platform conversion with intelligent dry and wet methods according to standard operating procedures (SOPs).

The particle grading curves of three soil samples were obtained, as shown in Figure 5d. The mass of 0.005~0.075 mm silt in soil samples exceeded 50% of the total mass of fine particles, and the mass of clay particles with a particle size of less than 0.005 mm was about 10%.

#### 2.2.2. Oil

Through environmental field investigation and geological survey, according to the composition analysis of soil samples, it is found that the leakage of waste cooking oil causes soil contamination. With the change in waste oil content, soil properties change. The mechanism of the influence of waste cooking oil infiltration on soil properties is still unclear, and there are few relevant studies. This paper carried out an experimental study on this topic. Since the main component of waste cooking oil is edible oil, light and low-viscosity vegetable oil was selected as the test oil. See Table 2 for its physical parameters.

#### 2.2.3. Prepare Oil Contaminated Soil

The test is divided into two groups, namely, a consolidated undrained test and a saturated seepage test. The effect of oil content on the mechanical properties of soil was studied by measuring the stress–strain relationship of soil samples through a consolidated undrained triaxial test. The saturated seepage test was used to study the influence of reducing the fine particle content in soil samples on the permeability coefficient and to explore the change in the permeability coefficient in the process of seepage deformation.

Soil samples with a dry density of 1.53 g/cm^3^, 1.68 g/cm^3^, and 1.79 g/cm^3^ and a grain size of 0.075~2 mm were each taken for preparing samples for the consolidated undrained test. The masses of soil, water, and oil were calculated according to Equations (1)–(3). The reconstituted soil samples were configured with a water content of 15%; an oil content of 0%, 3%, 6%, 9%, and 12%; a diameter of 3.8 cm; and a height of 7.6 cm. GDS triaxial apparatus (DYNTTS) was used for he consolidated undrained compression test.

(1) $$m_{0}=(1+0.01w_{0})\rho_{d}V$$

where $m_{0}$ denotes the mass of air dried soil, g; $w_{0}$ is air dried moisture content, %; $\rho_{d}$ is the dry density of soil sample, g/cm^3^; and V is the volume of soil sample compactor, cm^3^.

(2) $$∆m_{w}=0.01\left(w^{\prime }-w_{0}\right)\rho_{d}V$$

where $∆m_{w}$ is the amount of water that should be added to the soil sample, g, and $w^{\prime }$ is the water content to be configured for the soil sample, %.

(3) $$\Delta O=0.01\left(O^{\prime }-O_{0}\right)\rho_{d}V$$

where ΔO is the amount of oil that should be added to the soil sample, g; $O^{\prime }$ is the oil content to be configured for the soil sample, %; and $O_{0}$ is the initial oil content, which is generally zero.

### 2.3. Test Method

The soil sample was mixed with vegetable oil and left to stand for 24 h. The microstructure and main components of the soil sample were analyzed using scanning electronic microscopy (SEM) (Hangzhou, Zhejiang province, China) and energy-dispersive spectroscopy (EDS). Due to the poor conductivity of soil, it is necessary to perform sputtering with gold in advance, about 15 min before SEM scanning. After the sample stage is sent to the corresponding position of the instrument, the sample needs to be automatically evacuated for 20 min to avoid interference with the radiation signal of the scanned sample. A vacuum is maintained at 7.0 × 10^−5^ mbar, and then one moves to the sample stage using the relevant buttons and finds the best observation position, changes the magnification and focus, and adjusts the astigmatism until a satisfactory image is obtained. The voltage is 12~15 kV, and photos are taken at 1000 times magnification; then, satisfactory images are saved and printed.

The microscopic analysis adopts ZEIZZ-EVO15 (Hangzhou, Zhejiang province, China) scanning electron microscope for phase analysis, LaB6 electron gun, acceleration voltage EHT of 12~15 KV, probe current I probe of 20 pA, X-ray composition analysis current of 300~600 pA, and working distance WD of 8~15 mm. The EDS is used to determine the elemental composition of the sample by the back-scattered detector image. X-ray diffraction (XRD) was used to study the crystalline and amorphous phases of the original soil samples and the soil samples contaminated by vegetable oil.

**Figure 6:**
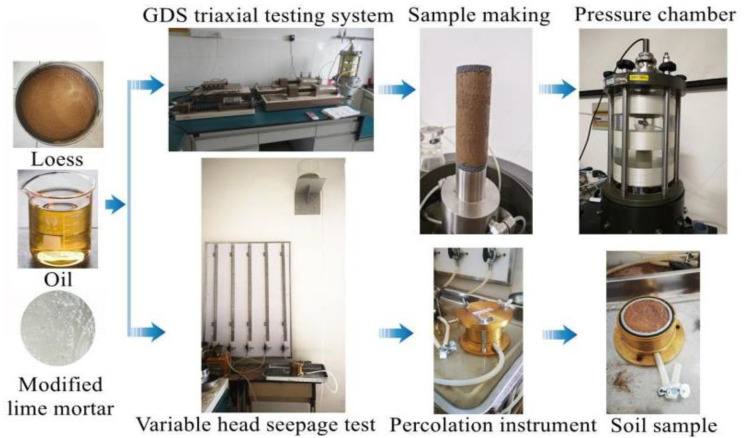
Schematic diagram of the experimentation process.

According to *Standard for geotechnical testing method (GB/T50123-2019)*, after evenly mixing with water, the soil surface is covered with film and left to stand at room temperature, 20 °C, for 24 h, and then the oil and soil are mixed with an oil content of 3%, 6%, 9%, and 12% and left to stand for 7 d. Oil content soil samples are prepared densities of 1.53, 1.68, and 1.79 g/cm^3^. After saturation in a vacuum in pure water in a vacuum cylinder for 8 h, the consolidated undrained test is carried out on the GDS triaxial tester. The test process is shown in Figure 6.

The MLM is then prepared. The main component of hydrated lime is Ca (OH)_2_, and the mineral powder is S95-grade ground granulated blast furnace slag. The modified lime mortar (MLM) is prepared according to a ratio of hydrated lime to mineral powder of 2:3, the water–cement ratio is 0.43, and additives such as viscosity additives are added. The modified lime mortar is mixed evenly with 5%, 10%, and 15% content and 6% oil-content-contaminated soil after 24 h of static and cured at 20 °C and 90% relative humidity for 7 days for standby.

See Table 3 for the composition ratio of the two test soil samples.

**Figure 7:**
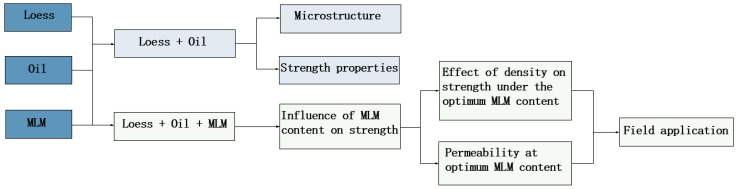
Flow chart of mechanical test for repairing oil-contaminated soil based on MLM.

The test flow of two soil samples is shown in Figure 7.

## 3. Experiment and Analysis

The test was divided into two groups. For the first group, the results of microstructure and strength properties of oil-contaminated soil with 0%, 3%, 6%, and 9% oil content were analyzed, and for the second group, the changes in strength and permeability properties of MLM were analyzed to improve oil-contaminated soil.

### 3.1. Characteristics of Oil-Contaminated Soil

#### 3.1.1. Microstructure Test

The microstructure and main constituent elements of soil samples were analyzed using scanning electron microscopy, and the microstructure of crystalline and amorphous materials of original soil samples and soil samples contaminated with vegetable oil were studied using phase analysis.

**Figure 8:**
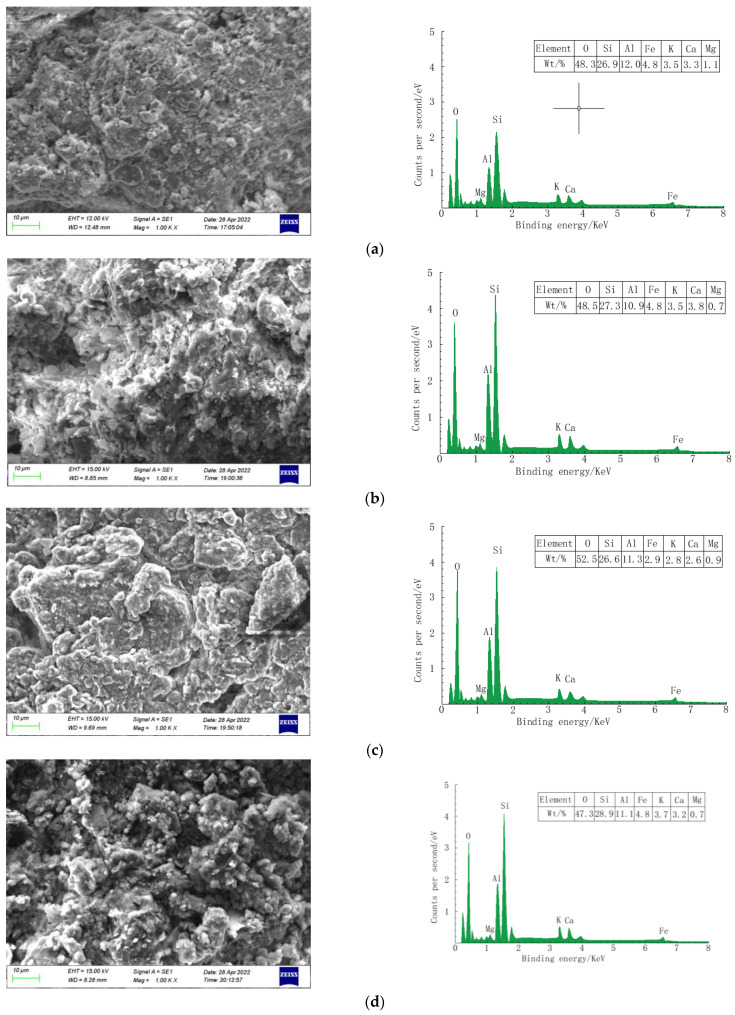
Microstructure and EDS of soil samples with oil content ($\rho_{d}$ = 1.68 g/cm^3^): (**a**) 0% oil content; (**b**) 3% oil content; (**c**) 6% oil content; (**d**) 9% oil content.

According to the image of the change in the SEM microstructure in Figure 8, it can be seen that the microstructures of silty clay with various oil content exhibit different characteristics. From Figure 8a, it can be observed that the microstructure of silty clay without oil is mainly granular. Figure 8b displays that the soil with an oil content of 3% is mainly scaly and massive. Figure 8c shows that when the oil concentration reaches 6%, the silty clay has a clear internal morphology of distinct flaky and block structures. The pores decrease and become compact. Figure 8d shows that silty clay with 9% oil content is granular, and small pores are obviously developed in it. In summary, energy spectrum analysis shows that the soil sample is mainly composed of O, Si, and Al and a small amount of Fe, K, Ca, and Mg. In the process of increasing the vegetable oil content, silty clay presents different structural properties. The undisturbed soil is powdery, and the scale-and-plate structure appears after the oil content increases. When the oil content continues to increase, the soil structure appears as porous cementation structures.

**Figure 9:**
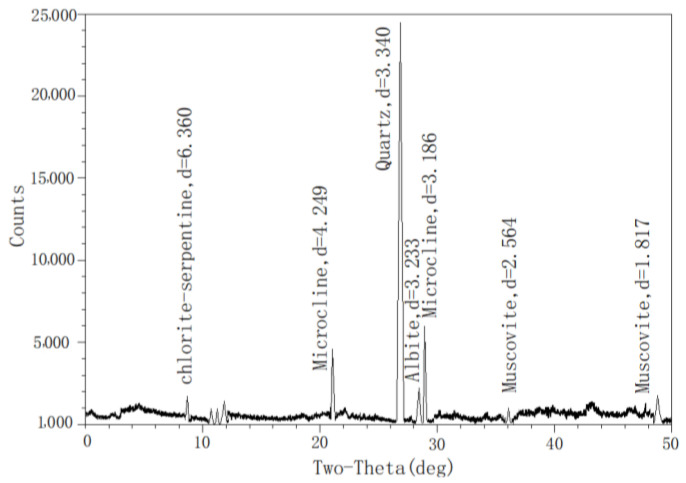
Mineralogical composition of silty clay sample at X-ray diffraction (XRD) test.

XRD diffraction analysis showed that there was no obvious change before versus after the crystal composition of the soil were contaminated by oil. It can be judged that there was no chemical reaction between vegetable oil and the soil, and no new substances were produced. Figure 9 shows that the main components of oil-contaminated soil samples were quartz (SiO_2_), microcline (KAlSi_3_O_4_), muscovite (K_0.77_Al_1.93_(Al_0.5_SiO_3.5_)(OH)_2_), Albite (AlSi_3_O_4_) and chlorite-serpentine ((Mg, Al)_4_(Si, Al)_4_O_10_(OH)_4_). Among these, quartz accounted for 48%, microcline accounted for 24%, muscovite accounted for 17%, albite accounted for 8%, and chlorite-seripentine accounted for 1%.

#### 3.1.2. Strength Property Test

When preparing soil samples, the dry density was 1.68 g/cm^3^, the water content was 15%, and the oil contents of the different samples were 0%, 3%, 6%, and 9%. The soil volume, water volume, and oil volume were measured according to Equations (1)–(3). The soil particles were weighted, and the water and oil were fully mixed and then covered with film for storage and left to stand at room temperature for 24 h. After the soil, water, and oil were evenly mixed, the soil was taken for sample preparation. After the sample was saturated in a vacuum for 7 h in the saturator, the GDS triaxial apparatus was used for a consolidated undrained compression test. The system shall set the confining pressure and back pressure by levels, and the confining pressure and back pressure differences of the different groups were 30 kPa, 60 kPa, 90 kPa, and 120 kPa. The pressure difference was kept between the confining pressure and, the backpressure was kept as a constant in each test. After each stage of confining pressure and backpressure pressurization, a pore pressure coefficient B value test was conducted. The pore pressure coefficient B was the pore-pressure coefficient under the condition of equal isotropic stress, which is the increment of pore pressure caused by the unit increment of confining pressure when the soil is in the state of isotropic compressive stress. If the B value is greater than or equal to 0.95, the sample can be considered saturated. Under special circumstances, the B value of individual samples remains unchanged for a long time, which is also considered saturation. After consolidation, the next undrained shear test can be conducted. When the bias stress reaches its peak value, 3~5% of the axial strain is then sheared. If the stress reading does not decrease significantly, it is sheared until the axial strain reaches 15~20%, and the whole test is complete. The peak value of deviatoric stress is taken. If there is no obvious peak value, the deviatoric stress corresponding to the axial strain of 15% is taken as the peak value.

**Figure 10:**
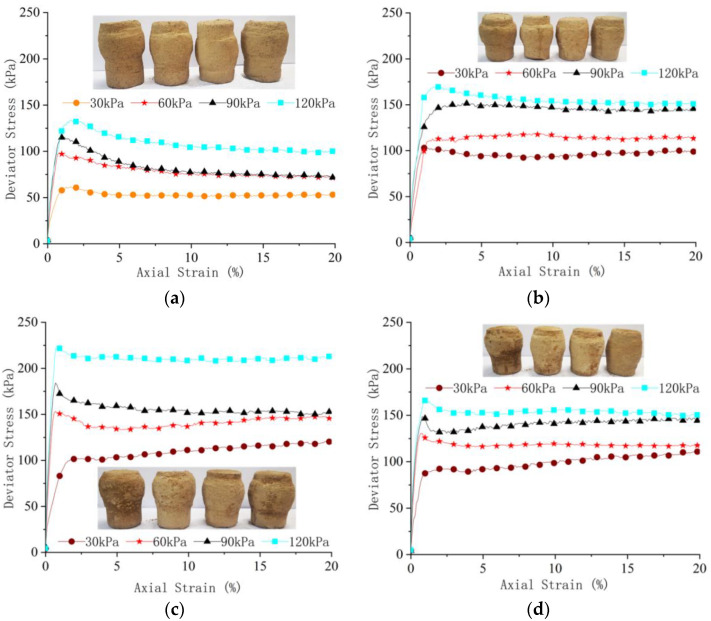
Relationship between principal stress deviation and strain: (**a**) 0% oil content; (**b**) 3% oil content; (**c**) 6% oil content; (**d**) 9% oil content.

Five parallel tests on each group of samples were carried out. Due to the influence of uncertain factors in the test, the data with large deviations were removed, the average values of 3–4 parallel test data were taken as the result; the deviatoric stress axial strain curves are drawn in Figure 10.

**Figure 11:**
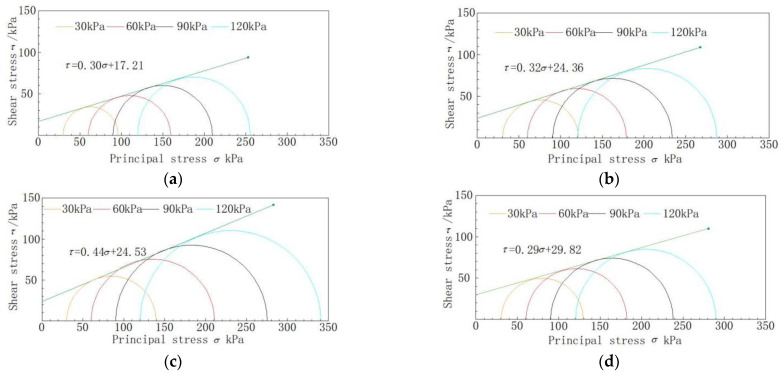
Mohr’s circle for stresses and strength envelope of four oil contents of soil samples ($\rho_{d}$ = 1.68 g/cm^3^): (**a**) 0% oil content; (**b**) 3% oil content; (**c**) 6% oil content; (**d**) 9% oil content.

It can be seen from Figure 10 that the peak value of the principal stress deviation also increases with the increase in the confining pressure. When the oil content is 6%, the peak value of the principal stress deviation reaches its maximum. The values under the four confining pressures are 110 kPa, 151 kPa, 185 kPa, and 221 kPa, which are 61.8%, 51.0%, 54.2%, and 62.5% higher than the oil-free soil samples, respectively. When the oil content increases to 9%, the peak value of the principal stress deviation of the soil sample decreases, showing a weakening trend of strength. It can be seen that with the increase in oil content in soil samples, the peak value of principal stress deviation first increases and then decreases, reaching an extreme value when the oil content is 6%. The total stress failure circle is drawn according to the peak value of principal stress deviation under each confining pressure, and the strength envelope of each stress circle is drawn. Soil samples with a density of 1.79 g/cm^3^ are taken to draw stress circle strength envelope lines with oil contents of 0%, 3%, 6%, and 9%, as shown in Figure 11. The dip angle and intercept of the envelope correspond to the friction angle and cohesion of soil samples, respectively. The friction angle and cohesion of soil samples with oil content are shown in Table 4. The friction angle of the soil sample increased with the increase in oil content. When the oil content increased to 6%, the friction angle reached a maximum value of 23.8° and then decreases. The cohesive force of the soil sample basically increased with the increase in oil content. The mechanical indicators of the soil show hardening.

### 3.2. Properties of MLM with Oil-Contaminated Soil

#### 3.2.1. Effect of MLM Content on Strength Index

In view of the large strength index of soil samples with an oil content of 6% and a density of 1.79 g/cm^3^, the change in the strength index of oil-contaminated soil after treatment with MLM content was tested. The MLM accounted for 0%, 5%, 10%, and 15%, respectively, and the soil samples with 6% oil content were prepared. The consolidated undrained test was completed after 7 days of curing under standard conditions. Five parallel tests were carried out on each group of samples, the average value of 3–4 parallel test data were taken as the result, and the molar stress circle and strength envelope were drawn; see Table 5 for its strength index. It can be seen that for the soil sample with 1.68 g/cm^3^ and an oil content of 6%, the friction angle gradually increased with the increase in the lime content. When the MLM content was 10%, the friction angle reached a maximum value of 30.07°, which is 1.26 times the friction angle of the non-lime-content-contaminated soil, and the maximum value of cohesion was 32.47 kPa, which was 1.32 times that of the non-lime content contaminated soil.

#### 3.2.2. Impact of Contaminated Soil Density on Strength Index

**Figure 12:**
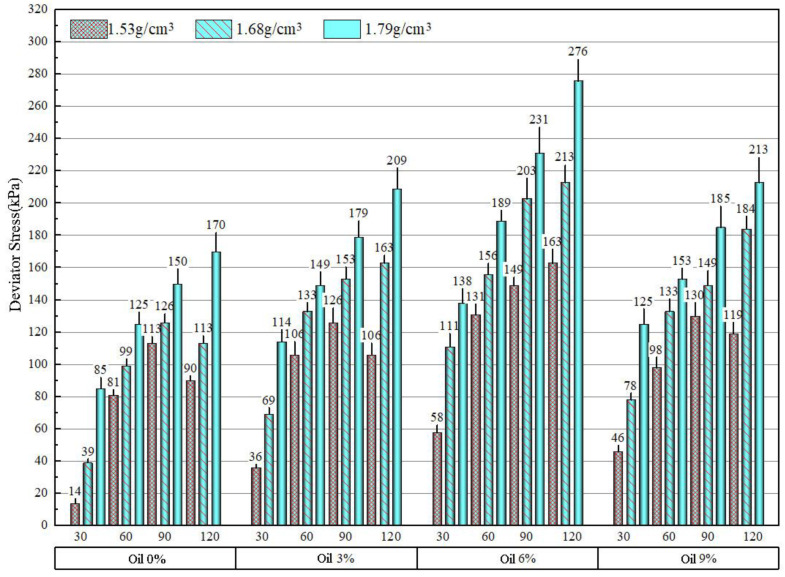
The mean values of the peak value of the principal stress deviation of three densities of MLM soil samples under different oil contents.

According to 10% of MLM content, soil samples with densities of 1.53 g/cm^3^, 1.68 g/cm^3^, and 1.79 g/cm^3^ were prepared. After the soil samples were cured under standard conditions for 7 days, five parallel tests and consolidated undrained shear tests were conducted on each group of samples, and the average value of 3–4 parallel test data was taken as the result. The peak value of the average principal stress deviation at each oil content is shown in Figure 11. The average friction angle and cohesion at each oil content are shown in Figure 12. It can be seen that the friction angle of the three densities of soil samples is the largest when the oil content is 6%, and the larger the density is, the larger the friction angle is, which is 25.59°, 27.72°, and 30.07°, with an increasing ratio of 8.3% and 8.5%.

**Figure 13:**
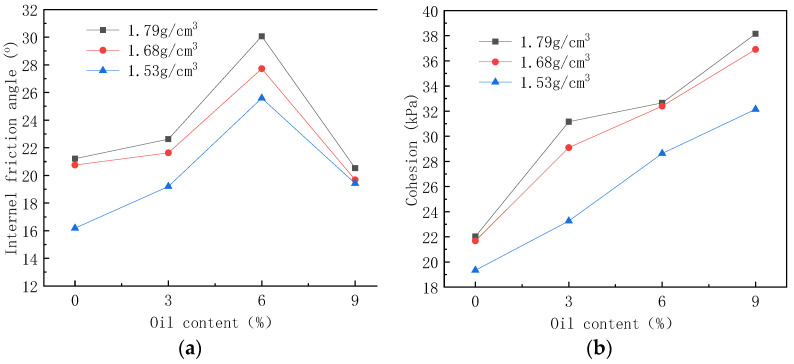
Strength index of three densities of MLM soil samples under each oil content (MLM content 10%). (**a**) Relationship between friction angle and oil content; (**b**) relationship between cohesion and oil content.

The peak value of the principal stress deviation of soil samples with densities of 1.53 g/cm^3^, 1.68 g/cm^3^, and 1.79 g/cm^3^ at each oil content is shown in Figure 12. The friction angle and cohesion at each oil content are shown in Figure 13. The cohesion of the three densities f soil samples increased with the increase in oil content. When the oil content was 9%, the cohesion was divided into 26.47 kPa, 27.67 kPa, and 29.82 kPa. The cohesion of the three densities of soil samples increased with the increase in oil content. When the oil content was 6%, the cohesion was divided into 28.63 kPa, 32.40 kPa, and 32.66 kPa.

#### 3.2.3. Permeability Test

(1) Influence of oil content on permeability

The permeability characteristics of soil samples with different oil content and 10% MLM content were studied. After the soil sample was saturated in a vacuum for 12 h, the permeability coefficient of the soil sample was measured using the variable-head permeability test. According to Darcy’s law, the seepage flow through the soil sample at the same time was equal to the flow through the water head gauge. The seepage coefficient can be expressed as

(4) $$k_{T}=2.3\frac{\mathrm{aL}}{A\left(t_{2}-t_{1}\right)}\log\frac{H_{1}}{H_{2}}$$

where a denotes the sectional area of variable head pipe, a = 3 cm^2^; L is seepage path, L = 4 cm; A is the cross-sectional area of the soil sample, A = 30 cm^2^; $t_{1}$, $t_{2}$ are the starting and ending time of the measured water head, s; and $H_{1}$ and $H_{2}$ are the starting and ending head, cm.

**Figure 14:**
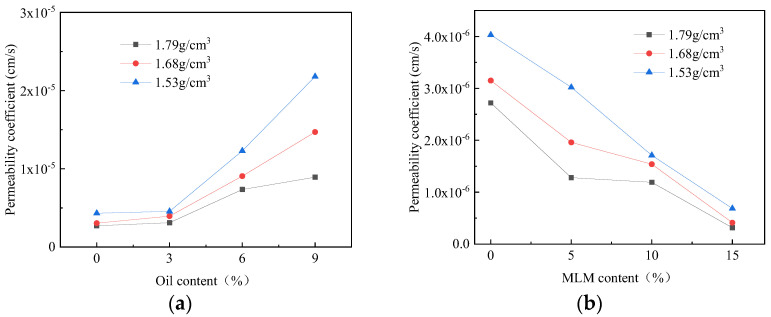
Permeability coefficient variation of three densities of soil samples. (**a**) Relationship between oil content and permeability coefficient (MLM content 10%); (**b**) relationship between MLM content and permeability coefficient (oil content 6%).

The relationship between the permeability coefficient of the three densities of soil samples and the oil content is shown in Figure 14a. The permeability coefficient increases with the increase in the oil content. The maximum increase was in the soil samples with a density of 1.53 g/cm^3^. The permeability coefficient of the soil samples with an oil content of 9% was 2.18 × 10^−5^ cm/s, which is 2.4 times the permeability coefficient of the 1.79 g/cm^3^ soil sample. The soil sample used in this test was silty clay mixed with MLM. After vegetable oil pollution, a film is formed on the surface of soil particles, which shortens the seepage path of water between soil particles and increases the permeability coefficient.

(2) Influence of MLM content on permeability

The permeability coefficient of the three densities o soil samples decreases gradually when the MLM content increases, and the relationship between the MLM content and the permeability coefficient is shown in Figure 14b. The permeability coefficient of the soil sample with a density of 1.53 g/cm^3^ and an MLM content of 15% was 9.86 × 10^−7^ cm/s, which is 24% of the permeability coefficient of soil samples with an MLM content of 0%, namely 4.03 × 10^−6^ cm/s.

The permeability coefficient of the three densities of soil samples decreased gradually with the increase in lime content, and the relationship between MLM content and permeability coefficient is shown in Figure 14b. The permeability coefficient of the soil sample with a density of 1.53 g/cm^3^ and MLM content of 15% was 9.86 × 10^−7^ cm/s and that of the soil sample with MLM content of 0% was 4.03 × 10^−6^ cm/s; the former is 24% of the latter.

## 4. Numerical Simulation Results and Discussion

### 4.1. Numerical Simulation Results

**Figure 15:**
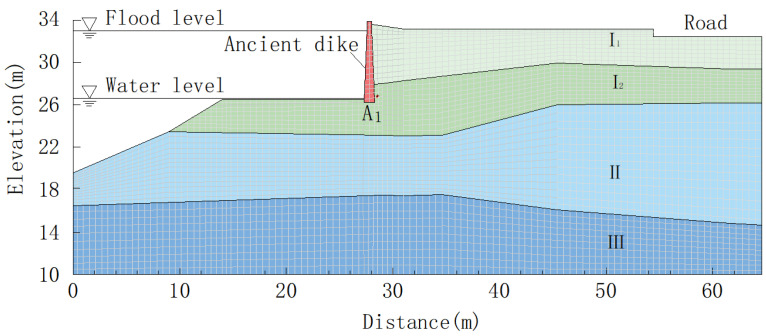
Numerical model of Lanxi ancient dike.

After the soil of the Lanxi ancient dike was contaminated with oil, the permeability coefficient and strength index of the soil changed, affecting the permeability deformation and stability of the bank slope. When using the finite element method to establish a numerical model [61], several diagonal lines are used to simplify the slope surface of the flood-controlled ancient dike. The elevation of the ancient dike top is 33.05 m, the elevation of the dike foot is 26.88 m, the elevation of the ancient dike foundation is 24.80 m, the height of the vertical section is 6.17 m, the width of the top is 0.22 m, the bottom height of the lower slope section of the upstream is 19.05 m, and the slope from bottom to top are 22° and 43°, respectively. The ancient dike is adjacent to the building, and the building foundation is a deep foundation. The upper load directly acts on the bearing layer, and its effect on the ancient dike can be ignored. The numerical model adopts quadrilateral continuous elastic-plastic elements with a grid edge length of 0.5 m, generating a total of 7023 nodes and 6877 elements. The numerical model of the ancient dike is shown in Figure 15. Point A_1_ is located 2.10 m above the foundation behind the ancient dike, that is, at 26.90 m elevation. See Table 6 for mechanical parameters of ancient dike soil after oil pollution and MLM repair.

**Figure 16:**
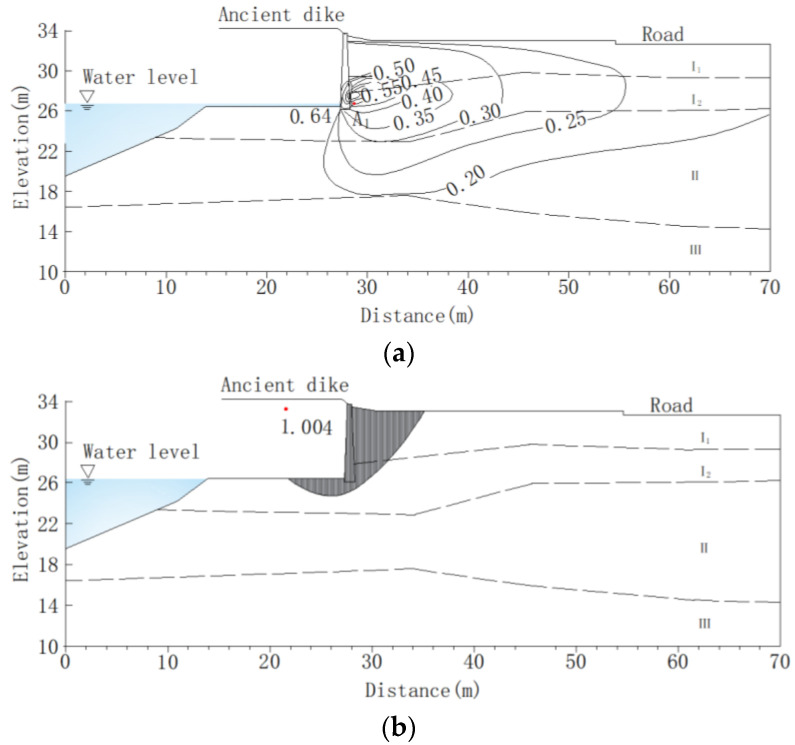
Analysis of seepage and bank slope stability of oil contaminated soil in ancient dikes (oil content of 6%). (**a**) Hydraulic gradient distribution map; (**b**) The minimum safety factor of bank slope stability and sliding surface.

**Figure 17:**
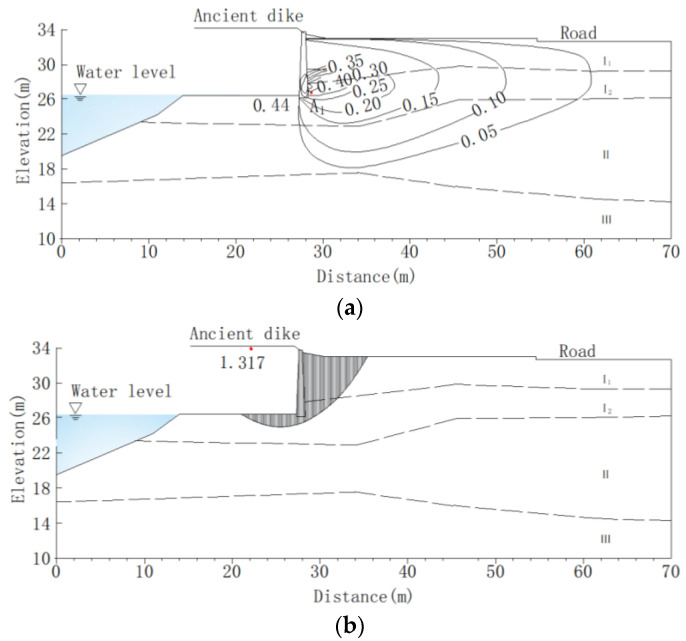
Analysis of seepage and bank slope stability in MLM repair of ancient dikes (MLM content 10%). (**a**) Hydraulic gradient distribution map; (**b**) The minimum safety factor of bank slope stability and sliding surface.

The numerical analysis results indicate that the seepage and slope stability of the ancient dike after being contaminated with oil are shown in Figure 16. Under external water level fluctuations, the hydraulic gradient of the soil varies with the location. Analyze the hydraulic gradient changes at point A_1_ on the back of the wall. The results of finite computing show that the hydraulic gradient of the ancient dike soil changes with the rise and fall of the flood peak of the Lanjiang River. At the maximum rate of water pressure change, the hydraulic gradient of the soil reaches its maximum value. The distribution of gradient values corresponding to a 6% oil content in the soil is shown in Figure 16a. When the water level change rate continues to increase, the hydraulic gradient at point A_1_ rapidly increases. When the water level is 26.58 m, the water level change rate reaches its maximum value, and the hydraulic gradient at point A_1_ reaches its maximum value of 0.64. When the water level change rate decreases, the hydraulic gradient begins to decrease. As the growth rate of the Lanjiang River water level slows down, the hydraulic gradient at point A_1_ gradually decreases. When the flood peak rapidly decreases, the hydraulic gradient decreases rapidly. When the water level changes slow down, the hydraulic gradient changes gradually stabilize. In the case of a sudden drop in water level in the Lanjiang River, when the rate of change reaches the stagnation point, the minimum stability safety coefficient of the ancient dike is 1.004. The typical calculated sliding surface is shown in Figure 16b. When using MLM to repair oil contaminated soil, when the MLM content is 10%, the permeability coefficient and mechanical parameters of the soil are changed, and the hydraulic gradient at point A_1_ decreases to 0.44, which is 31.3% lower than the hydraulic gradient value of oil contaminated soil. The minimum stability coefficient of the bank slope is 1.317, which is 31.2% higher than the stability safety coefficient of oil contaminated soil. As shown in Figure 17.

### 4.2. Repairing the Flood-Controlled Ancient Dike

**Figure 18:**
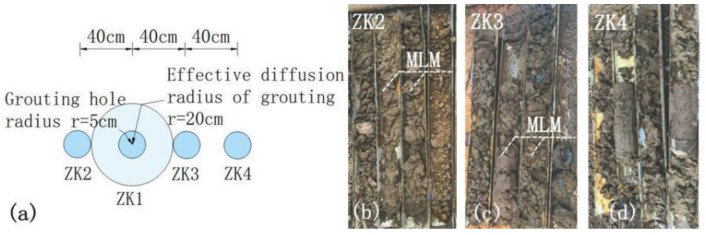
Grouting test of MLM. (**a**) Layout of grouting holes and inspection holes; (**b**) ZK2 borehole soil sample; (**c**) ZK3 borehole soil sample; (**d**) ZK4 borehole soil sample.

The MLM pressure grouting measures were adopted to repair the oil-contaminated soil in the ancient dike. A modified lime grouting test was conducted on the ancient dike slope, and ZK1 grouting holes were set up with a diameter of 10 cm. The MLM shall be grouted in six steps. The MLM shall be prepared according to the proportion, and then the soil surface shall be treated, the position of the grouting nozzle shall be calibrated, and then the hole shall be drilled. After the grouting nozzle is installed, the pressure grouting shall be carried out, and the static curing shall be more than 24 h. After the joint is sealed, the secondary grouting shall be carried out, and the pressure shall be controlled at 0.1~0.5 MPa, so as to ensure that the slurry is full without leakage and dense without bubbles. Seventy-two hours after the completion of ZK1 grouting, inspection boreholes were set up at positions ZK2, ZK3, and ZK4 with a hole spacing of 40 cm, as shown in Figure 18a. After testing the drilled soil samples, it was determined that the ZK2 and ZK3 drilling soil samples were filled with MLM, and no MLM was found in the ZK4 drilling soil samples, as shown in Figure 18b–d. Therefore, the effective diffusion radius of MLM was determined as 20 cm.

**Figure 19:**
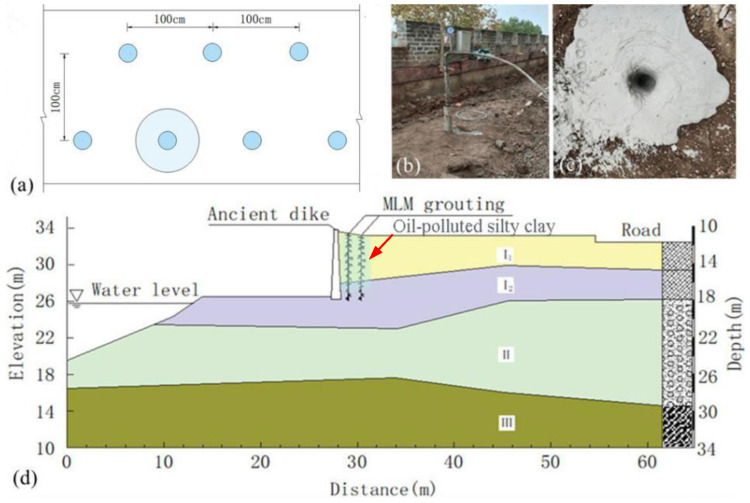
Repair measures of MLM pressure grouting: (**a**) distribution of grouting holes; (**b**) pressure grouting; (**c**) completion of grouting; (**d**) position and depth of pressure grouting.

The layout of MLM pressure grouting is shown in Figure 19a, divided into two rows with a spacing of 100 cm and a spacing of 100 cm between each row of holes. Considering the effective diffusion radius of the grouting as 20 cm, the MLM content in the MLM pressure grouting area can be calculated to be about 14.6%. The drilling process is shown in Figure 19b,c. The drilling depth was 6.00 m, as shown in Figure 19d.

After using MLM pressure grouting for ancient dikes, the pore water pressure and seepage ratio decreased significantly compared to the original structure section with good permeability. The results indicate that setting MLM pressure grouting can effectively reduce the permeability of the foundation soil, improve the cohesion of soil particles, and suppress the occurrence of piping in flood-controlled ancient dikes and repair engineering without changing the cultural relics. The compactness of the soil was improved inside the city wall, defects were treated, and leakage channels were blocked.

**Figure 20:**
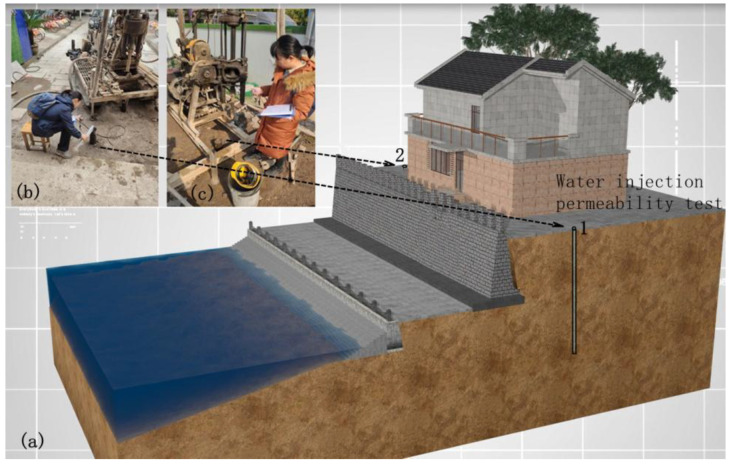
On-site water-injection permeability test. (**a**) Arrangement of water-injection holes; (**b**) water-injection test for hole 1; (**c**) water-injection test for hole 2.

In order to verify the anti-seepage effect of MLM pressure grouting, on-site water injection permeability tests were conducted, with two inspection holes arranged as shown in Figure 20. The results of the water injection permeability test are shown in Table 7. According to the test, the permeability coefficient of the soil after pressure grouting with MLM was reduced by 8~15%.

## 5. Conclusions

The physical properties, strength index, and permeability coefficients of oil-contaminated silty clay have changed, leading to the susceptibility of flood-controlled ancient dikes to disasters such as leakage and bank-slope instability. Through dynamic triaxial tests and variable head permeability tests, the strength index and permeability coefficient changes in oil-contaminated soil and MLM-improved soil were obtained. By using the finite element numerical simulation method for comparative analysis, the effect of MLM improvement on oil-contaminated soil on improving the leakage of flood-controlled ancient dikes and bank slope stability was obtained. Subsequently, MLM pressure grouting technology was used to repair the ruins of flood-controlled ancient dikes. The following conclusions were drawn from the above series of studies.

(1) After the silty clay was contaminated with oil, the relationship between the principal stress deviation and strain of the soil sample changed with the change in oil content, leading to stress hardening. When the oil content increased, the strength index also changed. When the oil content was 6%, the friction angle reached its maximum value, but as the oil content continued to increase, the friction angle decreased. When MLM was used to repair oil-contaminated soil, as the MLM content increased, the friction angle and cohesion of the soil sample also increased. When the MLM content was 10% and the oil content was 6%, the friction angle of the soil sample reached its maximum value. At the same MLM content, the higher the density of the soil sample, the greater the friction angle and cohesion, and the smaller the permeability coefficient.

(2) The finite element numerical simulation analysis shows that after the soil of the flood-controlled ancient dike was contaminated with oil, when the water level was at its maximum rate of change, and the water level was 26.58 m, the extreme hydraulic gradient of the ancient dike soil reached 0.64, and the extreme safety coefficient of bank slope stability was 1.004. When MLM was used to repair oil-contaminated soil, when the MLM content was 10%, the extreme hydraulic gradient of the ancient dike was 0.44, a decrease of 31.3%, and the extreme safety factor of bank slope stability was 1.317, an increase of 31.2%.

(3) MLM pressure-grouting technology was used to repair flood-controlled ancient dikes. The effective diffusion radius of MLM was detected through grouting tests, and then the distance, arrangement, and depth of MLM pressure grouting holes were determined. After the MLM grouting repair of oil-contaminated soil on the slope of the ancient dike was completed, on-site water injection permeability tests showed that the permeability coefficient of the ancient dike soil decreased by 8–12%.

Notably, this study can provide research methods and technical measures for the repair of similar flood-controlled ancient dikes.

## Figures and Tables

**Table 1:** Physical property parameters of silty clay.

Soil	Dry Density /g·cm^−3^	Volumetric Water Content/%	Liquid Limit /%	Plastic Limit /%	Plasticity Index	Specific Gravity
Ⅰ_1_	1.53 ± 0.08	44.4 ± 2.1	44.4	27.4	17.6	2.70
Ⅰ_2_	1.68 ± 0.07	41.2 ± 1.5	43.5	26.2	18.4	2.65
Ⅱ	1.79 ± 0.13	38.6 ± 1.1	/	/	/	2.72

**Table 2:** Physical parameters of edible oil (normal temperature) [59,60].

Oil	Density/g·cm^−3^	Viscosity Coefficient/Pa·s	Surface Tension/mN·m^−1^	Freezing Point/°C
Vegetable oil	0.912	11.2	34.2	−5.0

**Table 3:** Test soil sample grouping and sample preparation ratio.

Soil	Dry Density/(g/cm^3^)	Water Content/%	Oil Content/%	MLM Content/%
LO	1.53, 1.68, 1.79	15 ± 1	0, 3, 6, 9	0
LOM	1.53, 1.68, 1.79	15 ± 1	6	5, 10, 15

Note: LO is a mixed sample of silty clay and oil; LOM is a mixed sample of oil-contaminated silty clay treated with MLM.

**Table 4:** Strength index of soil samples with oil content (1.68 g/cm^3^).

Oil Content/%	Coulomb Line	Friction Angle/°	Cohesion/kPa
0	τ=0.30σ+17.21	16.79	17.21
3	τ=0.32σ+24.36	17.90	24.36
6	τ=0.44σ+24.53	23.80	24.53
9	τ=0.29σ+29.82	16.25	29.82

**Table 5:** Strength index of soil samples with MLM content (1.68 g/cm^3^, oil content 6%).

MLM Content/%	Coulomb Line	Friction Angle/°	Cohesion/kPa
0	y=0.44x+24.53	23.80	24.53
5	y=0.48x+26.71	25.84	26.71
10	y=0.57x+32.47	30.07	32.47
15	y=0.55x+32.32	29.03	32.32

**Table 6:** Physical and mechanical indexes of soil layer in the typical section.

No.	Name	Thickness /m	Dry Density /kN·m^−3^	Friction Angle (Oil Content 6%)/º	Cohesion (Oil Content 6%)/kPa	Initial Permeability Coefficient (Oil Content 6%)/cm·s^−1^	Friction Angle (MLM Content 10%)/º	Cohesion(MLM Content 10%)/kPa	Initial Permeability Coefficient (MLM Content 10%)/cm·s^−1^
Ⅰ_1_	Artificial fill-1	3.5	15.3	22.4	18.5	1.24 ×10^−5^	26.5	22.3	1.83 ×10^−6^
Ⅰ_2_	Artificial fill-2	4.1	16.8	23.8	24.5	9.70 ×10^−4^	30.1	32.5	1.65 ×10^−6^
Ⅱ	Sandy gravel	10.6	17.9	35.0	0	4.12 ×10^−4^	35.0	0	4.12 ×10^−4^
Ⅲ	Pelitic siltstone	>7	20.3	40.0	56.5	/	40.0	56.5	/

**Table 7:** List of water injection permeability test results of inspection holes 1 and 2.

No.	Oil-Contaminated Soil Test Results				MLM Grouting Repair Test Results
	Section	Hole Depth /m	Length /m	Permeability Coefficient/cm·s^−1^	Permeability Coefficient/cm·s^−1^
1	1	1.0–4.0	3.00	2.47 × 10^−4^	2.11 × 10^−4^ (↓14.6%)
	2	4.0–8.0	4.00	6.21 × 10^−5^	5.29 × 10^−4^ (↓14.8%)
	3	8.0–12.0	4.00	2.43 × 10^−4^	2.38 × 10^−4^
	4	12.0–16.0	4.00	1.11 × 10^−4^	1.09 × 10^−4^
2	1	1.0–4.0	3.00	2.15 × 10^−4^	1.96 × 10^−4^ (↓8.8%)
	2	4.0–8.0	4.00	1.67 × 10^−4^	1.46 × 10^−4^ (↓12.6%)
	3	8.0–12.0	4.00	1.58 × 10^−4^	1.52 × 10^−4^
	4	12.0–16.1	4.10	1.40 × 10^−4^	1.39 × 10^−4^

## Data Availability

Not applicable.
